# Understanding of Health literacy among Healthcare students in Saudi Arabia: a cross-sectional study

**DOI:** 10.1186/s12909-024-06263-8

**Published:** 2024-11-06

**Authors:** Naji Alqahtani, Adel Bashatah, Saeed Asiri, Wajid Syed, Mahmood Basil A. Al-Rawi

**Affiliations:** 1 Department of Nursing Administration and Education, College of Nursing, King Saud University, Riyadh, Saudi Arabia; 2 Department of Clinical Pharmacy, College of Pharmacy, King Saud University, Riyadh, 11451 Saudi Arabia; 3 Department of Optometry, College of Applied Medical Sciences, King Saud University, Riyadh, Saudi Arabia

**Keywords:** Literacy, Knowledge, Practice; handwashing; Hygiene, Healthcare students

## Abstract

**Background and objective:**

Health literacy is the cognitive and social abilities that influence a person’s motivation and capacity to learn, comprehend, and use information to improve and maintain good health. We aimed to investigate Healthcare Students’ knowledge, attitudes, and practices regarding Health Literacy in the Riyadh Province of Saudi Arabia.

**Methods:**

This study used prevalidated electronic questionnaires among healthcare undergraduates from colleges affiliated with Saudi University in Riyadh, Saudi Arabia, from December to April 2024. Healthcare students were asked to answer a self-designed online questionnaire sent to them through social media platforms.

**Results:**

The response rate was 560 out of 600 (93.3%); 293 of the 560 students (52.3%) were aged between 23 and 24 years (mean age was 21.29 SD = 1.852). The mean GPA among the students was 4.31(± 0.682). The average overall score for health literacy among healthcare students was 100.92 ± 11.80. The mean scores for knowledge were 24.17 ± 4.67, attitudes were 38.65 ± 4.20, and practice in health literacy was 38.09 ± 5.09. Among all healthcare students, 50.2% reported good knowledge, 54.1% had good attitudes, and 50.1% exhibited good practices in health literacy. However, students’ knowledge (*p* = 0.019) and training (*p* = 0.024) in health literacy were significantly associated with smoking cigarettes. Additionally, smoking shisha was significantly associated with students’ knowledge (*p* = 0.022), attitudes (*p* = 0.001), and practices (*p* = 0.001) in health literacy.

**Conclusion:**

Our study highlights that half of the healthcare students reported limited knowledge, attitude, and practice in health literacy. Age, gender, and course of study did not show significant differences. Rather, students who did not smoke cigarettes or shisha were found to have higher knowledge, attitudes, and practice in health literacy compared to smokers. However, to improve health literacy, additional education and increased awareness are needed. Further investigation is warranted to address the factors related to poor health literacy.

## Introduction

To safeguard, advance, and preserve optimal human health and well-being, health literacy is crucial [1, 2]. In light of the various pandemics, individuals are now more concerned about their health, which can be achieved by raising awareness of the issue or obtaining adequate education about it [3]. This highlights the importance of individuals, as well as healthcare professionals, to possess creativity, clinical expertise, and social responsibility in various health-related issues [4]. Insufficient health literacy is now a global concern for both the public and healthcare professionals [4, 5]. Health literacy is defined by the World Health Organization (WHO) as cognitive and social skills that impact an individual’s motivation and ability to acquire, understand, and apply knowledge to improve and maintain their health [4, 5]. Conversely, health literacy refers to a person’s ability to access, comprehend, and utilize basic health information or services to make informed decisions about their health [5].

Although health literacy is diverse and depends on the population being studied and the country of residence [2, 6, 7]. For example, in Europe, approximately 48% of them reported low health literacy [8], while among Chinese university students it was reported higher levels, for instances Wu et al. reported that 60.8% of Chinese students have insufficient health literacy [6], on the other hand, 63.5% of the Spain and France Health and Social Care University Students reported limited health literacy [2]. Furthermore, a recent systematic review of cross-sectional studies using a total of twenty-one research studies, indicates that university students’ health literacy scores are lower than those of reference samples [7]. In Saudi Arabia the prevalence of inadequate health literacy was 54.4% [7], while moderate health literacy was reported among 52.2% of nursing students, and 8.9% of the students in Saudi Arabia reported poor HL [1].

Insufficient health literacy occurs when the healthcare system fails to fulfill the requirements of individuals and patients [9]. As a consequence, the goals of developing HL include safe drug use, improved health outcomes, and appropriate use of health resources and services [10]. The incorporation of HL statistics into healthcare provider accreditation is a promising policy instrument for promoting HL principles in healthcare systems [1, 9, 10]. Research has also shown that various attributes of healthcare students, such as age, gender, course of study, parental education, and socioeconomic background, have a significant correlation with health literacy [1, 11]. In addition, Qadhi et al. (2023) state that healthcare professionals help patients discover possible interactions between the medications they are taking, to reduce the likelihood of drug-related problems, which lead to life-threatening situations [1]. In addition, reducing health inequality requires raising health literacy levels among healthcare undergraduates, as they are future practicing professionals. It is essential to provide effective, reliable, and readily available health information that meets the needs and circumstances of patients. Undergraduates are a vital source of future medical professionals who will safeguard the health and well-being of patients and other individuals, and serve as a backup force for the public health system. Evaluating undergraduates’ levels of health literacy and offering focused health education and hygiene education can assist in identifying and managing risky factors that endanger health and safety. To cope with this, healthcare professionals need to have strong health literacy. Therefore, this study aimed to assess the Healthcare Student’s Knowledge, attitudes, and practice towards Health Literacy at King Saud University, Riyadh, Saud Arabia.

## Methods

### Design, setting, participants, and data collection

A cross-sectional survey was conducted at a university in Riyadh, Saudi Arabia between January and April 2024. Before the study, the protocols were reviewed and approved by the Ethics Committee for Human Research at King Saud University in Riyadh, Saudi Arabia, under reference number (24–405). The study included undergraduates studying medicine, pharmacy, nursing, emergency medical services, and dentistry. Students from the fourth and fifth years were randomly selected for participation. The study population was targeted, and a senior researcher from the College of Pharmacy was assigned to collect data using computerized questionnaires created with Google Forms. Healthcare undergraduates completed the questions independently and anonymously. Before data collection, all healthcare undergraduates provided informed consent. The methods used in this investigation adhere to the principles of the Helsinki Declaration on human subjects research.

### Sample size

Similar to previous studies the sample size was calculated using an online calculator namely Raosoft [12–15] at a 5% margin of error and 95% confidence intervals. The total number of healthcare undergraduate students pursuing their third, fourth, fifth, and final year was 1600. As a result, the required sample size for this study was 310, however, we collected data from 600 students who were present during the data collection period.

### Questionnaire design

The questionnaires were developed after conducting a thorough review of similar studies on the topic [1, 6]. The questionnaire consisted of two parts [1, 6]. The questionnaire consisted of two parts. The first part included sociodemographic information such as age, gender, professional classification, year of study, presence of chronic disease, smoking status (including water pipes or shisha), and Grade Point Average (GPA). The second part was the “Five Characteristics Rating Scale” with 8 items, adapted from previous studies conducted by our research team [1, 6]. The third part of the study gathered information on the attitudes of healthcare students toward health literacy (9 items) [1, 6]. The final part of the study focused on the practice of health literacy, consisting of a total of 9 items. All items were assessed on a five-point rating scale ranging from Strongly Agree to Disagree [1, 6].

**Fig. 1:**
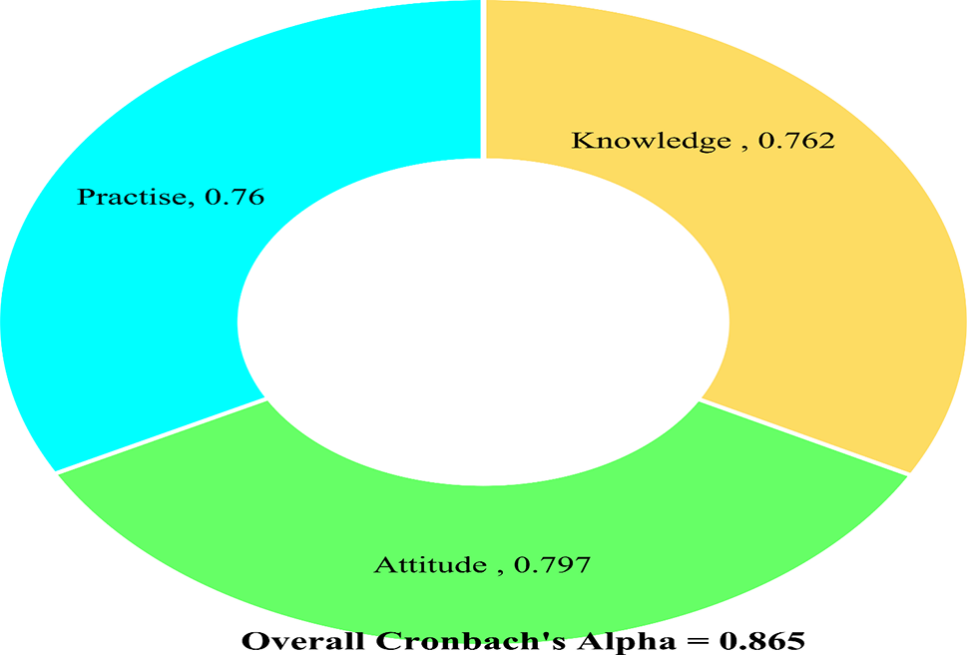
Cronbach’s Alpha for each scale in Health literacy

After the initial draft of the questionnaires, the questions were translated into Arabic using a forward and backward approach. The translated version was then reviewed by department specialists, including a researcher and a professor, to ensure its accuracy in Arabic. A pilot study was conducted with thirty randomly selected students before data collection to assess the appropriateness and flow of the questionnaires. The internal consistency was evaluated using Cronbach’s alpha, which was found to be 0.86, indicating that the questionnaires were valid and reliable for use in the study. Figure 1 displays the Cronbach’s Alpha value for each scale.

We utilized a scoring system where 1 indicated “strongly disagree” and 5 indicated “strongly agree.” Each item’s score was tallied, and the total scores for each domain (knowledge, attitudes, and practices) were calculated. The results were then divided into two categories: good (those who scored above 50%) and poor (those who scored below 50%). This approach was applied to the three domains: knowledge, attitudes, and practices. Next, the overall mean score for health literacy was determined by combining the mean scores of each domain (knowledge, attitudes, and practices) to arrive at the total mean score for health literacy. Additionally, the overall health literacy score was categorized as either adequate (for those who scored > 80% of the total mean score) or inadequate health literacy (for those who scored < 80% of the total mean score).

### Data analysis

The statistical package for social science (SPSS) version 27 (IBM, Armonk, NY, USA) was utilized for data analysis. Continuous variables were presented as mean ± standard deviation, while categorical variables were represented using frequency (n) and percentages (%). To investigate the relationship between healthcare students’ health literacy levels (domain-specific and overall) and demographic characteristics, a Chi-square/Fisher exact test was employed. Pearson correlation (r) was utilized to assess the relationship between health literacy domains. A p-value of < 0.05 was considered indicative of a statistically significant difference.

## Results

Five hundred and sixty students completed the survey. Among the respondents, the majority of them had a mean age of 21.29 ± 1.852 and 60.7% (*n* = 340) of them were female while 229(40.9%) were medical students. Around 120 (21.4%) of the students were in the second year and 18.2% (*n* = 102) were in the fourth year. The majority of the students claimed that they did not have any chronic disease. Regarding smoking habits 88.2% (*n* = 494) never smoked cigarettes while 86.4% (*n* = 484) never smoked water pipe tobacco. Table 1 contains the demographics and other characteristics of the study subjects.

**Table 1:** Healthcare student’s demographic, health, and other social characteristics (*n* = 560)

Characteristics	Frequency *n*	Percentage %	Mean (S.D)
**Age (Years)** 23–24 25–26 27	293 239 28	52.3 42.7 5.0	21.29 (± 1.852)
**Gender** Male Female	220 340	39.3 60.7	
**Professional** Pharm D Emergency medical services(EMS) Nursing Medicine Dentistry	130 36 133 229 29	23.2 6.5 23.8 40.9 5.2	
**Do you have any chronic disease**? Yes No	58 502	10.4 89.6	
**Ever smoked cigarettes**? Never smoked Former smoker Current smoker	494 41 25	88.2 7.3 4.5	
**Ever smoked water pipe tobacco**? Never smoked Former smoked Current smoker	484 45 31	86.4 8 5.5	
Your GPA* GPA of 0–2 GPA of 3–4 GPA of 5	08 121 47	1.4 21.6 8.4	4.31 (± 0.682)

The study found that 85.9% (*n* = 481) of healthcare students agreed or strongly agreed that if they were bitten or scratched by a dog or cat, they would clean the wound right away and get vaccinated against human rabies. Additionally, 73.6% (*n* = 412) of healthcare students strongly agreed that they were aware that chronic illnesses like HIV, hepatitis B, and hepatitis C are spread through mother-to-child contact, blood, and sexual contact but not through regular or occupational contact. Nonetheless, 20.4% (*n* = 114) of the students only agreed about it. Furthermore, according to the study findings, the majority of students 89.5% (*n* = 501) are aware that good hand hygiene and washing can help avoid influenza. In addition, 85.9% (*n* = 481) of healthcare students said that it is everyone’s responsibility to notify the CDC of any infectious illness cases that occur in their neighborhood. A very good understanding of physical activity was reported by the students, with 93.8% (*n* = 525) of them knowing that taking 6,000–10,000 steps a day is beneficial to one’s general health. Table 2 provides the specific frequency of the knowledge items.

**Table 2:** Healthcare Students’s responses towards knowledge of health literacy (*n* = 560)

Variables	Strongly agree *n*(%)	Agree *n*(%)	Neutral *n*(%)	Disagree/Strongly disagree *n*(%)
If a dog or cat bites or scratches, I’ll clean the wound right away and get vaccinated against human rabies.	365(65.2%)	116(20.7%)	52(9.3%)	27(4.8%)
I am aware that daily or occupational contact does not spread the human immune deficiency virus, hepatitis. Rather, these diseases are transmitted by mother-to-child contact, sexual contact, and blood.	412(73.6%)	114(20.4%)	25(4.5%)	9(1.6%)
I am aware that good hand hygiene and washing can help stave off the flu.	397(70.9%)	104(18.6%)	49(8.8%)	10(1.8%)
I think it is everyone’s responsibility to notify the CDC of any infectious illness cases that occur in their community.	363(64.8%)	118(21.1%)	71(12.7%)	8(1.4%)
I know that eating a light diet, with less oil, salt, and sugar is beneficial for health.	372(66.4%)	137(24.5%)	32(5.7%)	19(3.4%)
I am aware that it is against the law to conceal my travels, start an epidemic, or “fail to attend an organized nucleic acid test” without a valid reason.	371(66.3%)	145(25.9%)	43(7.7%)	1(0.2%)
I am aware that walking 6,000–10,000 steps a day is beneficial to your health.	416(74.3%)	109(19.5%)	32(5.7%)	3(0.5%)
I am aware that taking the right precautions lowers the risk of getting HIV and STIs as well as avoiding unintended pregnancies.	374(66.8%)	137(24.5%)	45(8%)	4(0.7%)
I keep and treat raw and cooked food separately, wash raw vegetables and fruits, and avoid eating rotten or expired food.	362(64.6%)	152(27.1%)	28(5%0	18(3.2%)

When it comes to attitudes toward health literacy, 77.7% (*n* = 435) of healthcare students agreed or strongly agreed that they do not use communal spoons and chopsticks when eating with friends and family. Additionally, 70% (*n* = 392) agreed or strongly agreed that they keep common medical and first aid equipment at home. Furthermore, 58% (*n* = 325) of healthcare students believed that if they experienced anxiety, depression, dread, despair, or other psychological difficulties during a severe infectious disease pandemic, they would handle them on their own. The majority (95.4%; *n* = 534) of healthcare students also agreed or strongly agreed to actively assist medical and health staff in carrying out emergency measures such as investigation, isolation, disinfection, and immunization, as shown in Table 3.

**Table 3:** Healthcare student’s attitudes towards health literacy (*n* = 560)

Variables	Strongly agree *n*(%)	Agree *n*(%)	Neutral *n*(%)	Disagree/ Strongly disagree *n*(%)
When I eat with friends and family, I don’t use shared spoons or chopsticks.	327(58.4)	108(19.3%)	82(14.6%)	43(7.7%)
I keep common medical and first-aid materials at home.	252(45%)	140(25%)	92(16.4%)	76(13.6%)
During an infectious illness outbreak, I feel it’s important to handle any psychological issues, like anxiety, depression, dread, and despair, independently.	213(38%)	112(20%)	106(18.9%)	129(23%)
I believe that the decision to seek medical treatment for an infectious condition is personal and that others have no right to intervene.	321(57.3%)	73(13%)	62(11.1%)	104(18.6%)
I will actively collaborate with medical and health staff to implement emergency measures such as inquiry, isolation, disinfection, and immunization.	393(70.2%)	141(25.2%)	24(4.3%)	2(0.4%)
I refuse to smoke or spit in public, and if I cough or sneeze, I cover my mouth and nose.	470(83.9%)	78(13.9%)	10(1.8)	2(0.4%)
I will carefully study the package, labels, and directions before purchasing food, medicine, or health items.	303(54.1%)	128(22.9%)	111(19.8%)	18(3.2%)
I learn about the outbreak through official media reporting and do not listen to alternative sources.	339(60.5%)	128(22.9%)	63(11.3%)	30(5.4%)
I can identify common hazard indicators such as high pressure, flammability, explosiveness, highly poisonous, radioactivity, biosecurity	318(56.8%)	171(30.5%)	48(8.6%)	23(4.1)

The findings of the study suggest that healthcare students demonstrate good health literacy practices. For instance, when asked about checking body temperature, 60.7% (*n* = 340) of students agreed or strongly agreed that they would shake the thermometer to below 35 °C, place it under their armpit against the skin, and remove it after 5 min to take a reading. When it comes to checking blood pressure, 73.8% (*n* = 413) of students agreed or strongly agreed that wrapping the cuff around the elbow 2–3 cm above the heart and placing it as tightly as possible to fit one finger in is the first step. In the event of an earthquake, 77.7% (*n* = 435) of healthcare students agreed that if they are working or studying in a building, they should take the lift and evacuate as quickly as possible. The detailed frequencies of health literacy practices among healthcare students are provided in Table 4.

**Table 4:** Healthcare Student’s Practice toward Health Literacy (*n* = 560)

Variables	Strongly agree *n*(%)	Agree *n*(%)	Neutral *n*(%)	Disagree/Strongly disagree *n*(%)
To check temperature, it needs to shake below 35 °C, press it against at skin beneath the armpit, and then take it out five minutes later to get a temperature reading.	211(37.7%)	129(23%)	154(27.5%)	66(11.8%)
To measure the blood pressure first it involves wrapping the cuff around the elbow, 2–3 cm above the heart, and pressing it firmly enough to accommodate one finger.	280(50%)	133(23.8%)	97(17.3%)	50(8.9%)
If there is an earthquake when I’m studying or working in a building, I’ll use the elevator to get out of there as soon as I can.	373(66.6%)	62(11.1%)	31(5.5%)	94(16.8%)
If there is a flood, I will evacuate according to the rules of moving people before objects and in a systematic manner.	318(56.8%)	172(30.7%)	44(7.9%)	26(4.6%)
I shall check for gas leaks by turning on the lights as soon as I get home from work if I detect a strong gas scent.	326(58.2%)	73(13%)	58(10.4%)	103(18.4%)
Chest compressions will be performed at a depth of 5–7 cm at a rate of 100–120 compressions per minute	227(40.5%)	171(30.5%)	132(23.6%)	30(5.4%)
To save the life of an electrocuted victim, I first turned off the power source without coming into contact with the victim.	385(68.8%)	116(20.7%)	50(8.9%)	9(1.6%)
I will place pieces of cloth or wood in the mouths of people who are close to me if they are having an epileptic fit so they can’t bite their tongue.	255(45.5%)	130(23.2%)	123(22%)	52(9.3%)
I will stay low, cover my mouth and nose with a damp towel, and phone the fire department or emergency services when fleeing a fire.	405(72.3%)	109(19.5%)	36(6.4%)	10(1.8%)

**Fig. 2:**
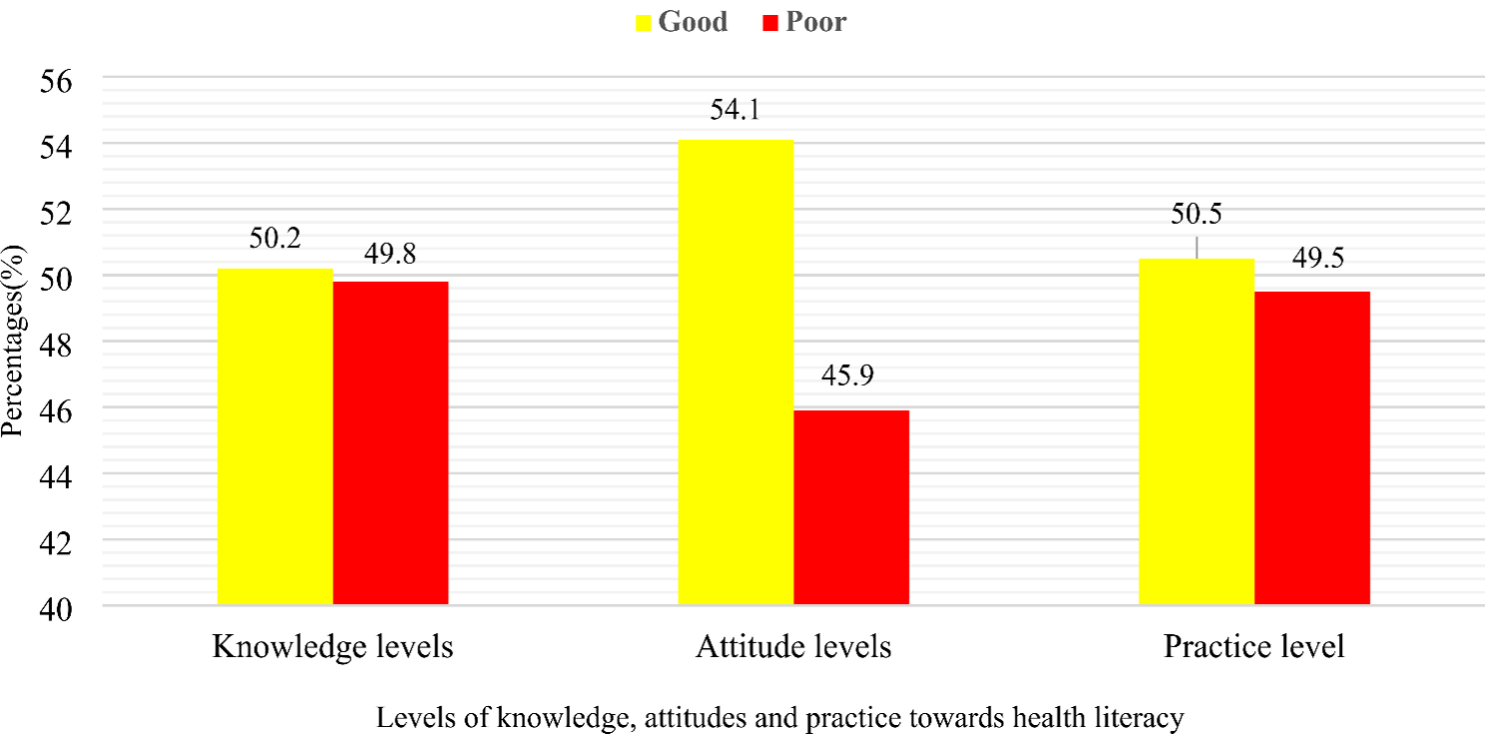
Levels of Knowledge, attitudes, and practice towards Health literacy

With regards to the domain of Health literacy, 50.2%(*n* = 281) of the healthcare students were found to have good knowledge of health literacy, while 54.1%(*n* = 303) of them reported good attitudes and 50.5%(*n* = 283) of good practice as shown in Figure 2.

The associations between students’ knowledge, attitudes, and perception scores with demographic factors such as gender, classification, GPA level, presence of chronic disease, smoking cigarettes, and smoking shisha were determined using Chi-square or Fisher exact tests, with *p* < 0.05 values considered significant. Gender was not significantly associated with knowledge, attitudes, or perception scores of health literacy, as indicated by p-values of *p* = 0.679, *p* = 0.117, and *p* = 0.078, respectively. However, students’ knowledge (*p* = 0.019) and practice (*p* = 0.024) in health literacy were significantly associated with smoking cigarettes. Additionally, smoking shisha was significantly associated with students’ knowledge (*p* = 0.022), attitudes (*p* = 0.001), and practices (*p* = 0.001) in health literacy as shown in Table 5.

**Table 5:** Association between Healthcare Students’ knowledge, attitudes and Practice of Health literacy and demographic characters

Variables		Knowledge		*p* value	Attitude		*p* *value*	Perception		*p* *value*
		Good	Poor		Good	Poor		Good	Poor	
**Gender** Male	Count	108	112	0.679	110	193	0.117	101	119	0.078
	% within sex	38.4%	40.1%		36.3%	63.7%		35.7%	43.0%	
Female	Count	173	167		110	147		182	158	
	% within sex	61.6%	59.9%		42.8%	57.2%		64.3%	57.0%	
**Classification** EMS	Count	14	17	0.302	18	13	0.060	17	14	0.101
	% within Classification	5.0%	6.1%		5.9%	5.1%		6.0%	5.1%	
Medicine	Count	127	104		136	136		112	119	
	% within Classification	45.2%	37.3%		44.9%	44.9%		39.6%	43.0%	
Nursing	Count	66	72		67	71		76	62	
	% within Classification	23.5%	25.8%		22.1%	27.6%		26.9%	22.4%	
Pharm D	Count	58	73		62	69		58	73	
	% within Classification	20.6%	26.2%		20.5%	26.8%		20.5%	26.4%	
Dental	Count	16	13		20	9		20	9	
	% within Classification	5.7%	4.7%		6.6%	3.5%		7.1%	3.2%	
**Grade point average(GPA)** **0–2**	Count	3	5	0.767	3	5	0.097	2	6	0.435
	% within GPA	3.4%	5.7%		2.8%	7.1%		2.4%	6.5%	
**3–4**	Count	61	60		79	42		58	63	
	% within GPA	69.3%	68.2%		74.5%	60.0%		69.9%	67.7%	
**5**	Count	24	23		24	23		23	24	
	% within GPA	27.3%	26.1%		22.6%	32.9%		27.7%	25.8%	
**Presence of chronic disease** Yes	Count	26	32	0.389	33	25	0.653	32	26	0.456
	% within chronic disease	9.3%	11.5%		10.9%	9.7%		11.3%	9.4%	
No	Count	255	247		270	232		251	251	
	% within chronic disease	90.7%	88.5%		89.1%	90.3%		88.7%	90.6%	
**Smoking cigarettes** Never smoked	Count	255	239	0.019	273	221	0.229	252	242	0.024
	% within smoking cigarettes	90.7%	85.7%		90.1%	86.0%		89.0%	87.4%	
Former smoker	Count	12	29		17	24		14	27	
	% within smoking cigarettes	4.3%	10.4%		5.6%	9.3%		4.9%	9.7%	
Current smoker	Count	14	11		17	12		17	8	
	% within smoking cigarettes	5.0%	3.9%		5.6%	4.7%		6.0%	2.9%	
**Smoking Shisha** Never smoked	Count	249	235	0.022	263	221	0.001	245	239	0.001
	% within smoking shisha	88.6%	84.2%		86.8%	86.0%		86.6%	86.3%	
Former smoker	Count	14	31		16	29		14	31	
	% within smoking shisha	5.0%	11.1%		5.3%	11.3%		4.9%	11.2%	
current smoker	Count	18	13		24	7		24	7	
	% within smoking shisha	6.4%	4.7%		7.9%	2.7%		8.5%	2.5%	

**Fig. 3:**
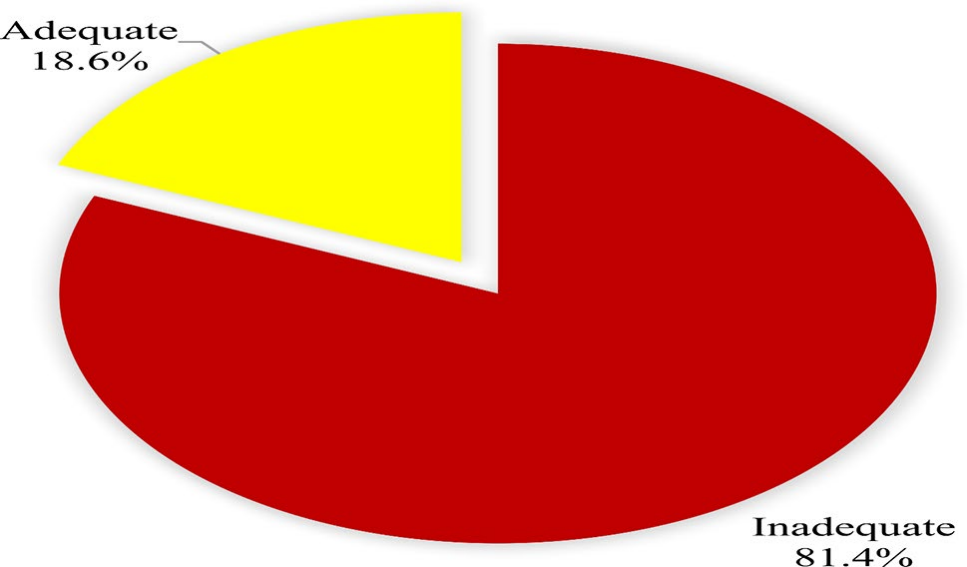
Overall health literacy levels among healthcare students

Furthermore, of the healthcare students 18.6%(*n* = 104) were classified as adequate and the majority 81.4%(*n* = 456) were inadequate in health literacy (Figure 3.

In this study, the mean score of knowledge of health literacy was 24.17 ± 4.67 (Median = 26.00; minimum = 10; Maximum 30; range 0–20), while the mean score for attitudes towards health literacy among healthcare students was 38.65 ± 4.20(median = 39.00; minimum 27; maximum 45, range 0–18). Similarly, the mean score for practice was 38.09 ± 5.09 (median 39.00; minimum 24; maximum 45; range 39.00). The mean overall score (knowledge, attitude, and practice) was 100.92 ± 11.80 (median 101.00; minimum 65.; maximum 119; range 0–54.). The associations between healthcare students’ health literacy levels with factors such as age, gender, year of study, classification, presence of chronic disease, smoking cigarettes and shisha, and GPA were determined using Chi-square and Fisher exact tests at the *p* < 0.05 level (Table 5). Results showed that gender and professional classification of the healthcare students were not found associated with health literacy levels, while the presence of chronic diseases(p = 0.089), smoking cigarettes (p = 0.008), and shisha (*p =* 0.001) was significantly associated with health literacy level. The associations between health literacy levels and healthcare students ' demographics are shown in Table 6.

**Table 6:** Association between Healthcare students’ health literacy levels and demographics

Variables		Overall score		Total	*p*-value
		Inadequate	Adequate		
**Gender** Female	Count	274	66	340	0.525
	% within sex	80.6%	19.4%	100.0%	
	% within overall two levels	60.1%	63.5%	60.7%	
Male	Count	182	38	220	
	% within sex	82.7%	17.3%	100.0%	
	% within overall two levels	39.9%	36.5%	39.3%	
**Classification** Emergency medical services(EMS)	Count	20	6	26	0.588
	% within professional Classification	76.9%	23.1%	100.0%	
	% within overall two levels	4.4%	5.8%	4.6%	
Medicine	Count	188	40	228	
	% within professional Classification	82.5%	17.5%	100.0%	
	% within overall two levels	41.2%	38.5%	40.7%	
Nursing	Count	112	21	133	
	% within professional Classification	84.2%	15.8%	100.0%	
	% within overall two levels	24.6%	20.2%	23.8%	
Pharm D	Count	104	26	130	
	% within professional Classification	80.0%	20.0%	100.0%	
	% within overall two levels	22.8%	25.0%	23.2%	
**Presence of chronic disease** Yes	Count	52	6	58	0.089
	% within chronic disease	89.7%	10.3%	100.0%	
	% within overall two levels	11.4%	5.8%	10.4%	
No	Count	404	98	502	
	% within chronic disease	80.5%	19.5%	100.0%	
	% within overall two levels	88.6%	94.2%	89.6%	
**Smoking cigarettes** Never smoked	Count	404	90	494	0.008
	% within smoking cigarettes	81.8%	18.2%	100.0%	
	% within overall two levels	88.6%	86.5%	88.2%	
Former smoker	Count	37	4	41	
	% within smoking cigarettes	90.2%	9.8%	100.0%	
	% within overall two levels	8.1%	3.8%	7.3%	
Current smoker	Count	15	10	25	
	% within smoking cigarettes	60.0%	40.0%	100.0%	
	% within overall two levels	3.3%	9.6%	4.5%	
**Smoking Shisha** Never smoked	Count	398	86	484	0.0001
	% within smoking shisha	82.2%	17.8%	100.0%	
	% within overall two levels	87.3%	82.7%	86.4%	
Former smoker	Count	41	4	45	
	% within smoking shisha	91.1%	8.9%	100.0%	
	% within overall two levels	9.0%	3.8%	8.0%	
current smoker	Count	17	14	31	
	% within smoking shisha	54.8%	45.2%	100.0%	
	% within overall two levels	3.7%	13.5%	5.5%	

The relationship between the three aspects of health literacy such as knowledge, attitude, and practice is demonstrated by the findings of the Pearson correlation analysis. Results of Pearson analysis indicated that knowledge and attitude had a correlation coefficient of *r* = 0.514, whereas knowledge and practice had a correlation coefficient of *r* = 0.512. All three of these dimensions were positively significant, and the correlation coefficient between attitude and practice was *r* = 0.683. In this context, the relationship between attitudes and practice indicates that close relation as shown in Table 7.

**Table 7:** Correlation matrix for, knowledge, attitude, and practice toward Health literacy

Variables	Knowledge	Attitude	Practice
Knowledge	1		
Attitude	0.514**	1	
Practice	0.512**	0.683**	1

Pearson’s correlation was used. **Correlation is significant at the 0.01 level.

## Discussion

In this study, 50.2% of healthcare students reported having good knowledge of health literacy, while 54.1% of them had good attitudes and 50.1% had good practices. In terms of overall health literacy, only 18.6% of students reported having adequate, while 81.4% of them reported in adequate health literacy. The majority of healthcare students in this study agreed that if they were bitten or scratched by a dog or cat, they would promptly clean the wound and receive a human rabies vaccination. They also showed good knowledge regarding the fact that chronic diseases such as HIV, hepatitis B, and hepatitis C are spread through mother-to-child contact, sexual contact, and blood, but not through everyday or workplace contact. In this study, 50.2% of the students demonstrated good health literacy. These results were in line with previous research conducted by Wu et al. in 2023 among university students and Qadhi et al. among nursing students. For instance, in the Wu et al. study the authors stated that students had a mean score of 4.36 and agreed to visit the hospital right away to rinse the wound and receive a human rabies vaccination as soon as feasible [6]. Qadhi et al.’s study shows that 38.9% of students were found to have good health literacy while 52.2% reported moderate health literacy [1]. Previous research found that university students’ lower health literacy is primarily determined by age, gender, the number of semesters completed, the course of study or curriculum, parental education, and socioeconomic background [7]. 

The overall health literacy knowledge, attitudes, and practice score in this study was 100.92 ± 11.80. In terms of the individual scale scores, the knowledge score was 24.17(4.67), the mean attitude score was 38.65(4.20), and the practice score was 38.09(5.09). Additionally, healthcare students with chronic diseases, smoking cigarettes, and using shisha were significantly associated with overall health literacy levels. These results are consistent with those of Wu et al. among Chinese university students, who reported a mean score of 105.33 ± 10.14 for health literacy and mean scores of (36.093 ± 4.192), (34.178 ± 4.227), and (35.059 ± 4.515) for knowledge, attitudes, and practices, respectively [6]. S Similar to previous findings, female students demonstrated higher health literacy levels than male students, and lower-grade students scored higher than higher-grade students. Students at metropolitan universities also exhibited higher health literacy levels than those at rural universities [6]. In this study, health literacy was not found to be associated with gender or classification of students. This disparity may be due to differences in educational systems and societal aspects. Additionally, health literacy can vary between studies and depends on factors such as the sample being studied, the types of questionnaires used, and methodological approaches.

Regarding attitudes, 54.1% of the students stated that they had positive attitudes and that they don’t share chopsticks or spoons when dining with friends and family. The majority of the students also reported having common first aid and medical items at home. These results were in line with previous findings [1, 6]. For example, an earlier study by Qadhi et al. among nursing students reported that Health literacy means being able to seek healthcare as soon as there is a health status change. In addition, most of the students agreed that Health literacy involves carrying out healthcare orders correctly outside of a health facility and it is the ability to adhere to treatment [1].

In addition, the findings of the research demonstrate that healthcare students are practicing good health literacy. For example, 60.7% of the healthcare students agreed that they would shake the thermometer below 35 °C, press it against the skin under the armpit, and then take it out after five minutes to get a reading. Similarly, the practice was good towards recording the blood pressure reading, where 73.8% of the students agreed that one finger should be able to pass through the cuff once it has been wrapped around the elbow, two to three cm above the heart, and firmly pressed. A previous study by Qadhi et al. reported that nursing students always exchange health-related information. In terms of medical checkups, one-third of the students always have yearly routine checkups. Furthermore, students reported always seeking medical attention when they are ill [1]. The variation in the current findings compared to earlier findings may be best explained by the fact that students in health-related programs are familiar with health-related knowledge, the healthcare environment, topics of health promotion, and disease prevention.

Current research suggests that in addition to understanding health literacy as future practitioners, healthcare students should also develop strong practical skills that can assist them in their practice and future careers. Good health literacy has been linked to positive health outcomes in previous studies [16–19]. Individuals with low health literacy tend to engage in unhealthy practices [20, 21]. This highlights the importance of not only having knowledge, attitude, and practice abilities related to health literacy as an individual but also understanding it as a prospective health professional. Healthcare students can therefore grasp the concept of health literacy and help patients develop healthy practices. According to the literature, there has been limited research evaluating the health literacy knowledge of Saudi healthcare students. There is insufficient national and international literature on the knowledge, attitudes, and practices of health literacy. However, most research reports on individual and public knowledge of health literacy and its practices [10, 11, 22–24]. The results of this study would make a significant contribution to the literature in Saudi Arabia and other countries, serving as a reference for future research. These results could be used by healthcare and educational institutions to develop training programs that effectively enhance healthcare workers’ clinically relevant health literacy.

## Limitations

This study has several limitations. To begin with, the information presented here may not accurately represent the knowledge of all Saudi Arabian undergraduate healthcare students studying healthcare, as it was limited to students from a single university in Saudi Arabia. Therefore, the findings cannot be generalized internationally or to other regions within Saudi Arabia. Additionally, social desirability bias may have been present due to the self-administered nature of the online questionnaire used to collect data. To enhance the validity of the findings, further research with larger sample sizes, including undergraduate healthcare students from various institutions in Saudi Arabia focusing on healthcare, is necessary.

## Conclusion

Our study highlights that half of the healthcare students reported limited knowledge, attitude, and practice in health literacy. Age, gender, and course of study did not show significant differences. Rather, students who did not smoke cigarettes or shisha were found to have higher knowledge, attitudes, and practice in health literacy compared to smokers. However, more education and sufficient awareness are required to promote health literacy. We suggest employing a greater number of participants in a subsequent study to assess health literacy and address the factors linked to low health literacy. The findings of this study effectively highlight the importance of health literacy in shaping the future practices of healthcare professionals. The recommendations for enhancing health literacy through education and awareness initiatives are well-founded and could inform policy and curriculum development in healthcare education.

## Data Availability

The data used for this study will be available from the corresponding author upon request.
